# The Role of the Negative 3-H Trio in Second Language Education

**DOI:** 10.3389/fpsyg.2021.736928

**Published:** 2021-08-18

**Authors:** Jing Chu, Xiaoxia Zeng

**Affiliations:** ^1^College of Literature, Bohai University, Jinzhou, China; ^2^School of International Communication, Dongguan Polytechnic, Dongguan, China

**Keywords:** negative 3-H trio, positive psychology, positive peace psychology, emotions, second language education

## Abstract

Language has been proven to strongly affect different aspects on one's life/career including his/her identity and interpersonal communication skills beyond the immediate context. Given this, now proper discourse and interlocutor's emotions are highlighted in academia. However, few studies (if any) have explored the role of negative stressors and constructs in L2 classroom discourse and interpersonal communication competency. To fill this yawning lacuna, the present study provided a glance at the impact of three negative language aspects of *hate, hurt*, and *harm* (also called negative 3-H trio) on L2 education. Moreover, it presents the definitions, origins (positive psychology, positive peace psychology), dimensions, and applications of each aspect. Finally, some implications and future directions are suggested to avid scholars in L2 and mainstream education.

## Introduction

Languages and words are by no means neutral tools but considerably powerful to exert short-term and long-term impacts on people's minds and hearts (Lewandowska-Tomaszczyk et al., 2020). They can permeate into one's identity, unite/split people, establish/remove boundaries, and simultaneously produce harmony and conflict (Siddiq, 2016). Language is at the core of human's natural quest for connectedness to a community in which the quality of relationships is largely dependent on one's interpersonal communication expertise. Language and discourse can form a harmonious context for living, working, and studying if the interactants are linguistically and (inter)culturally aware and competent (Holmes, 2008; Wang et al., 2016). Contrarily, improper use of language can ruin everything and generate conflict, violence, adversity, or even wars. Hence, it is conceivable that positive relationships and peace in any context depend on a chain of rings with language and communication skills being the core.

In foreign/second language education which is full of adversities, caring for stakeholders' emotions and interpersonal communication abilities is vital as they influence many aspects of teaching and learning including engagement, performance, achievement, well-being, motivation, and success (Gabryś-Barker, 2016; Derakhshan, 2021; Greenier et al., 2021; Xie and Derakhshan, 2021). All the desired outcomes of education are at the mercy of a positive atmosphere and rapport between the teacher and students in the class as a social context. This raises the significance of communication skills such as proper discourse, credibility, clarity, immediacy, and care for cultural disparities. Additionally, in EFL/ESL contexts in which the students grapple with a different language and culture, peace-building practices are pivotal. This conceptualization is engrained in *positive psychology* and *positive peace psychology* as two recent trends which focus on “how people thrive” and “actively build peace” instead of dwelling on life's negativities and inequalities (Gibson, 2011; MacIntyre and Mercer, 2014). However, these schools do not ignore the role of negative emotions and conflicts in teaching and learning.

In tune with this contention, the present article aimed to scrutinize three negative aspects of classroom language (i.e., hate, hurt, and harm) known as the 3-H trio, represented *via* a bad language and classroom context. In EFL contexts where teachers and students, sometimes, cross linguistic and cultural boundaries on some subjects, interpersonal communication knowledge and awareness are essential for practitioners to observe the pre-figured classroom objectives and, in turn, convert the negative aspects into peace-building activities which can upsurge several aspects of L2 education.

## Background

### Positive Psychology vs. Positive Peace Psychology

The interconnectedness of emotions, inner states, language, and education is best addressed in positive psychology (PP) and positive peace psychology (PPP) as two recent trends. These schools have commonalities yet function independently. PP examines how individuals can flourish and be happier by focusing on positive emotions like joy, hope, passion, resilience, optimism, and the like instead of negative feelings (Dewaele, 2015; MacIntyre et al., 2019; Li, 2020; Li et al., 2020; Greenier et al., 2021; Pishghadam et al., 2021). It rests on three pillars of *positive subjective experience* (emotions), *positive individual traits* (individual characteristics), and *positive institutions* (contexts) (MacIntyre and Mercer, 2014). On the other hand, PPP, which is fresher, capitalizes on how to vigorously establish peace and social justice instead of focusing chiefly on how to preclude or eradicate violence and conflict (Gibson, 2011). In L2 settings which are full of setbacks, PPP is a non-violent approach that underscores peace through peaceful tools to produce harmonious relationships (Gregersen and MacIntyre, 2021). Both trends run against dysfunctional, absence-based, and deficit-oriented conceptualizations of wellness and peace. Like PP which highlights positive emotions without disregarding negative stressors, PPP considers peace to go beyond the absence of conflict (Peterson, 2006).

### Interpersonal Communication and Peace in L2 Classrooms

By nature, the human being is social and seeks interpersonal, intergroup, and intercultural connections with others irrespective of physical and special proximity. According to socio-cultural theory (SCT) and the social dimension of constructivism, this creates a web of associations among people worldwide whose behaviors and actions leave strong imprints on others (Davies-Vengoechea, 2003). In the globalization era in which people from various cultures and social norms seek communication with others, interpersonal communication skills are of paramount importance to survive and convey the message properly. In the context of world Englishes and English as an international language (EIL), learning English is not just for classroom use but to have intercultural communications *via* scientific works, conferences, meetings, and so on. Hence, EFL/ESL and mainstream educational contexts need to develop interpersonal communication skills/awareness of students and teachers. This is the case as people belonging to different cultures may perceive the same thing differently. So, interpersonal interaction skills do not only take into account “what to say” but also about “how to say it” to observe intercultural norms and etiquettes. In L2 education in which stakeholders face numerous linguistic and intercultural conflicts, there must be formed a harmonious and peaceful environment for learning to occur. This needs a positive rapport and interpersonal competency to bear disparities and even convert them into learning opportunities.

**Figure 1:**
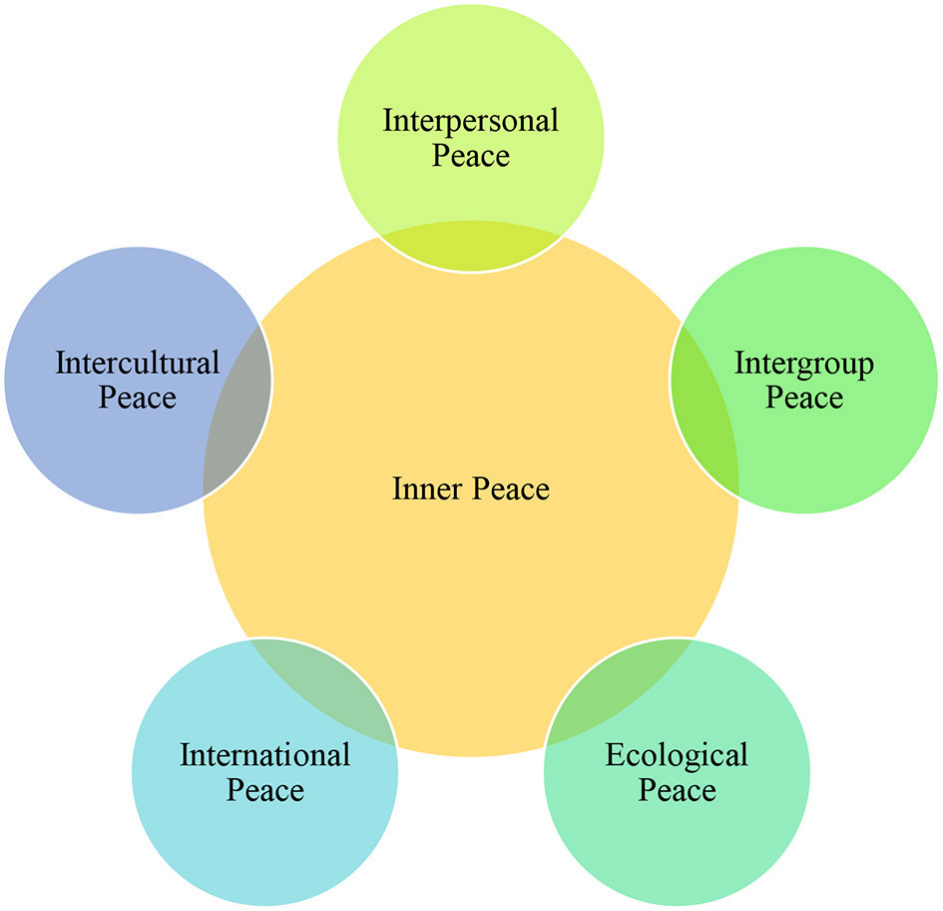
Dimensions of peace language.

As stated, people may think about an identical issue from different angles and form different opinions which may lead to conflict in L2 classrooms. What matters extensively in such unharmonious milieus is the importance of discourse and discursive devices used to express meanings. EFL practitioners as peace-builders should educate their students on the way they produce the language and its consequences. Other than meta-linguistic knowledge, EFL students need to know the dimensions of a peaceful language as well. According to Oxford (2014), peace has six nested dimensions including *inner, interpersonal, intergroup, intercultural, international*, and *ecological peace* (Figure 1).

*Inner peace* is the core dimension that concerns harmony in the heart and inside the individual. *Interpersonal peace* is harmony and caring for family and friends and includes love, trust, kindness, compassion, and respect. *Intergroup peace* is harmony occurring among groups classified by religion, race, gender, age, class, and ethnicity. *Intercultural peace* concerns harmony among different societies and cultures. *International peace* refers to international collaboration among different nations. Finally, *ecological peace* concerns valuing and caring for the natural environment. These are critical in L2 education as peace involves establishing a positive relationship with people belonging to other cultural groups, respecting their rights, and resolving conflicts constructively (Oxford, 2014). In the absence of classroom peace, interpersonal communication skills like interaction clarity, credibility, and immediacy which affect different aspects of learning are unlikely to emerge. Correspondingly, conflicts and disputes may pop out in a community of practice (COP) in which there is no or insufficient harmony among its members. Therefore, EFL teachers are obliged to develop their students' interpersonal communication skills to operate efficiently inside and outside their English class and offer useful peace-building activities to create a positive discourse context that generates favorable outcomes such as improved students' engagement, motivation, interest, achievement, resilience, and success.

### The Tripartite of Negative 3-Hs in Language Education: The Definitions and Applications

**Figure 2:**
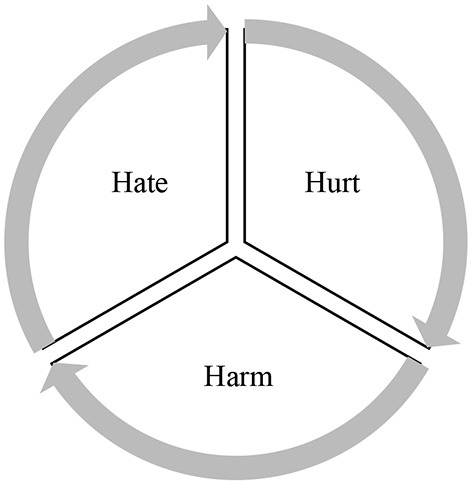
The negative 3-H trio of language.

The traditional myth that teaching and learning depend solely on teachers' pedagogical techniques and students' attempts is now dispelled with the emergence of emotionology in education. Given its prominence in EFL/ESL contexts, research on inner states, feelings, and emotional aspects of language learning and teaching has witnessed a boom of interest among scholars with the advent of humanistic psychology (Prior, 2019). These emotions can be negative (e.g., stress, anxiety, and tension) and positive (e.g., optimism, grit, hope, flow, and happiness). Concerning the role of positive emotions, numerous studies indicated that they increase engagement, motivation, achievement, success, efficacy, interest, performance, etc. (Bolkan, 2017; Derakhshan et al., 2019; MacIntyre et al., 2019; Li, 2020; Derakhshan, 2021; Wang and Derakhshan, 2021). As for negative emotions, a huge body of research points to their detrimental impacts on the teaching-learning cycle from different angles including motivation, passion, strategic-investment, retention, concentration, satisfaction, and performance. What seems to be missing in researching negative emotions in L2 learning which has been in the limelight for decades until the turn of the millennium and a shift of focus toward positive emotions introduced by PP is the role of negative factors and stressors in the classroom discourse and interculturality level of EFL students and teachers. As a case in point, the conceptualization and impact of negative language aspects like hate, hurt, and harm (known as negative 3-H trio) produced by language has long been kept under the carpet until Curtis and Oxford's 2021 groundbreaking study which defined the concepts and their practical applications in the classroom to establish a peaceful learning context. As the first H element, “hate” refers to a dislike feeling about someone or something which is the opposite of “love.” It can be expressed through different communicative modes (written, spoken) and causes destruction on various levels from personal (e.g., break up with someone) to societal level (initiating wars). On the other hand, “hurt” is the emotional and physical damage of the language to someone which can be transient and lasting depending on the severity of hateful language. The final H, “harm” seems much similar to “hurt” but its degree of injury is higher. Like “hurt,” “harm” causes both emotional and physical injury. It is worth noting that these triple Hs are by no means mutually exclusive and there are some overlaps and associations among them. To put it differently, they are like a nested system that has grown out of each other in that hateful language and discourse hurts and harms people. Likewise, hurting and harming others with hateful language generates and sparks hatred (Figure 2).

Another important note is that these three negative language aspects vary in their degree (i.e., slight, severe) and duration (i.e., short-term, long-term) depending on the discourse context and interlocutors. A hateful, hurtful, and harmful language or message may be damaging for an interlocutor in a specific context but bearable in another setting for another interlocutor. In L2 education which is imbued with cultural and linguistic adversities and the penultimate goal is to communicate effectively in the globalized world, stakeholders are required to know the role and significance of their discourse in the class. In a class made up of a group of students belonging to dissimilar cultures, EFL students and teachers are expected to use a positive and respectful language to create peace and harmony as prerequisites of learning and teaching. As the knowledge of interpersonal communication skills and the mentioned tripartite model is context and culture-specific, EFL practitioners need to turn the paradox of using the negative trio into reality by some instructional techniques. Teachers as the catalysts of change can do so by offering activities related to each H including running discussions in L1 about “bad language” and its dire consequences, using audio-visual tools (pictures, drawings, magazines, and cartoon) related to a hatful, hurtful, and harmful interpersonal communication behavior, using dictionaries and other primary sources to spot the origin and development of the three concepts, conducting critical discourse analysis (CDA) on films and textbooks representative of the negative 3-H tripartite, and discussing different impacts of a bad language on the process of language education.

In summation, the triple H model is very important in English language education as EFL/ESL students are now facing speakers from different cultures which entails improving their intercultural awareness/competence and minding their language as it might cause irretrievable damages in international encounters.

### Implications and Future Directions

In this article, it was contended that language affects many aspects of one's life including education and identity. Moreover, emotions and inner states were found to have a close tie with success in mainstream and L2 education. With the spread of English and the removal of temporal and special boundaries, now EFL/ESL students need to be experts in intercultural interaction norms which is not achievable except through an education that concerns the impacts of emotions on language utterances. Focusing on three negative constructs of hate, hurt, and harm, the present research went through the roots, definitions, applications, and dimensions of these language aspects. The results are insightful for EFL and mainstream education teachers and students in that they increase their awareness and competency in interpersonal communication skills and the power of language through appropriate classroom tasks. Teachers can develop their interpersonal skills to establish a friendly rapport in the class which facilitates the transmission of knowledge to the students. Moreover, teacher trainers can offer training programs and workshops to pre-service and in-service teachers regarding different language-related emotions and interpersonal communication skills. Furthermore, materials developers can benefit from this study in that they can design materials and tasks which reflect the negative 3-H trio and their criticality. Finally, researchers in L2, as a microcosm of general education, can conduct future studies on other emotion-related variables and their impacts on language education and classroom discourse. Avid researchers are also recommended to qualitatively explore this tripartite in mainstream education, EFL, and ESP contexts from the perspectives of different stakeholders. Finally, running CDA, case, and longitudinal studies through diaries and portfolios are novel ideas in this line of research as well.

## Author Contributions

JC: revised the majority of the original paper. XZ: added the figures and analyzed the figures. All authors contributed to the article and approved the submitted version.

## Conflict of Interest

The authors declare that the research was conducted in the absence of any commercial or financial relationships that could be construed as a potential conflict of interest.

## Publisher's Note

All claims expressed in this article are solely those of the authors and do not necessarily represent those of their affiliated organizations, or those of the publisher, the editors and the reviewers. Any product that may be evaluated in this article, or claim that may be made by its manufacturer, is not guaranteed or endorsed by the publisher.
